# Vitamin D Levels in Asymptomatic Adults-A Population Survey in Karachi, Pakistan

**DOI:** 10.1371/journal.pone.0033452

**Published:** 2012-03-23

**Authors:** Adil Sheikh, Zeb Saeed, Syed Ali Danial Jafri, Iffat Yazdani, Syed Ather Hussain

**Affiliations:** 1 Medical College, Aga Khan University Hospital, Karachi, Pakistan; 2 Nephrology, Department of Medicine, Aga Khan University Hospital, Karachi, Pakistan; University of Sao Paulo Medical School, Brazil

## Abstract

**Background:**

It is well established that low levels of 25(OH) Vitamin D (<30 ng/dL) are a common finding world over, affecting over a billion of the global population. Our primary objective was to determine the prevalence of vitamin D deficiency and insufficiency in the asymptomatic adult population of Karachi, Pakistan and the demographic, nutritional and co-morbidity characteristics associated with serum vitamin D levels.

**Methods:**

A cross-sectional population survey was conducted at two spaced out densely populated areas of the city. Serum levels of 25OHVitamin D were measured and GFR as renal function was assessed by using 4 variable MDRD formula.

**Results:**

Our sample of 300 had a median age of 48(interquartile range 38–55) years. The median level of serum vitamin D was 18.8 (IQ range 12.65–24.62) ng/dL. A total of 253 (84.3%) respondents had low levels (<30 ng/dL) of 25OH vitamin D. Serum PTH and vitamin D were negatively correlated (r = −0.176, p = 0.001). The median PTH in the vitamin D sufficiency group was 38.4(IQ range28.0–48.8)pg/mL compared with 44.4(IQ range34.3–56.8) pg/mL in the deficiency group (p = 0.011).The median serum calcium level in the sample was 9.46(IQ range 9.18–9.68) ng/dL. Low serum levels of vitamin D were not associated with hypertension (p = 0.771) or with an elevated spot blood pressure (p = 0.164).In our sample 75(26%) respondents had an eGFR corresponding to stage 2 and stage 3 CKD. There was no significant correlation between levels of vitamin D and eGFR (r = −0.127, p-value = 0.277).Respondents using daily vitamin D supplements had higher 25 OH vitamin D levels (p-value = 0.021).

**Conclusion:**

We observed a high proportion of the asymptomatic adult population having low levels of vitamin D and subclinical deterioration of eGFR. The specific cause(s) for this observed high prevalence of low 25OH vitamin D levels are not clear and need to be investigated further upon.

## Introduction

Vitamin D is one of the major players involved in calcium homeostasis in the human body. It has been shown that sufficient serum levels of vitamin D are needed for bone health and development. Vitamin D deficiency has been associated with development of osteoporosis due to induction of secondary hyperparathyroidism, which mobilizes calcium out of bones increasing the risk of fall related fractures especially in the elderly [1]. Other then decreased bone mineral density vitamin D deficiency can present as rickets and osteomalacia, in the pediatric and adult population respectively[2]. It has also been associated with myopathy and may hold key while addressing to unexplained aches and pains presentations in the outpatient setting [3].

Along with its proven beneficial role in bone mineralization, the vitamin has been described as having ‘steroid like action’ as it regulates the function of over two hundred genes [4]. Recent evidence has shown that vitamin D has a role as an immune modulator and tumor suppressor [5], [6], [7]. It has been proposed that this vitamin may help in prevention of chronic diseases including type 1 diabetes mellitus, coronary heart disease, multiple sclerosis, rheumatoid arthritis and cognitive impairment [8], [9], [10], [11], [12], [13], [14].

Low levels of vitamin D (<30 ng/dL) is a common finding world over [15], [16] and varies depending on the population studied, adherence to food fortification policies, demographic features, geographic location and season. It has been estimated that over one billion people globally have low serum vitamin D levels [17]. Majority of individuals with vitamin D deficiency/insufficiency are asymptomatic–making it a difficult clinical entity to detect. According to the clinical practice guidelines on vitamin D issued by Endocrine Society [18], vitamin D deficiency has been defined as serum 25(OH) vitamin D levels less than 20 ng/dL whereas insufficiency constitutes serum 25(OH) vitamin D levels between 20 ng/dL and 30 ng/dL. Serum levels greater than 30 ng/dL are deemed sufficient for children and adults.

25(OH) vitamin D is used as the marker of vitamin D status in the body, rather than 1, 25(OH) vitamin D which represents the active metabolite of vitamin D. Low levels of 1, 25(OH) vitamin D is an expected and commonly seen phenomenon in chronic kidney disease patients due to decreased 1-alpha hydroxylase activity, however the effect of CKD on levels of 25(OH) D is not as yet clear. A high prevalence of 25(OH)D deficiency has been observed in CKD patients in numerous studies and it has been seen that 25(OH) vitamin D deficiency worsens with progression of CKD, due to compromise in denovo synthesis of precursors of 25(OH) D [19], [20], [21]. Ravani ET. al. observed that 25(OH)D and plasma phosphate were independent predictors of renal disease progression and death in CKD patients [8].

The reported prevalence of low levels of vitamin D (<30 ng/dL) in our region ranges from 85–98%, as observed by numerous authors. A cross sectional study done in Karachi Pakistan, on employees in a tertiary care center revealed 90% of the employees having low vitamin D levels [17]. A 92% prevalence of vitamin D deficiency is reported by Zuberi et. al. in retrospectively studied asymptomatic ambulatory patients presenting to the endocrinology outpatient service in a tertiary care center in Karachi [22]. Similarly studies done in neighboring India report the prevalence of low levels of vitamin D to be ranging between 80–85% in groups of postmenopausal women and local hospital staff [23], [24]. Prevalence of Vitamin D deficiency and insufficiency was found out to be 80% in healthy adults living in urban Tehran, Iran [25].

Most of the published literature on Vitamin D levels in Pakistan is performed in smaller outpatient settings or single centers, involving specific groups and pockets of the population. There is a dearth of published data on prevalence of vitamin D deficiency in asymptomatic population of urban centers, such as Karachi. Therefore giving us a rationale to conduct a cross sectional study in densely populated areas of urban Karachi with the primary objective to estimate the prevalence of vitamin D deficiency and insufficiency in the asymptomatic population along with observing the associations between serum 25(OH) vitamin D levels and demographic, nutritional and co-morbidity characteristics of the population studied. Another aim of our study is to find any association of vitamin D levels with e GFR and CKD status. Since it has been seen that CKD may be a risk factor for vitamin D deficiency and that vitamin D deficiency is a predictor for renal disease progression in CKD, we tried to look at any associations between vitamin D status and eGFR in asymptomatic adults with subclinical deterioration in kidney function.

## Methods

### Ethics statement

Study methodology was approved by the Ethical review committee of the Aga Khan University(ERC protocol-1628). Informed consent was taken from respondents by means of an informed consent form. Participants were informed regarding their laboratory results by means of postal service.

### Setting

The study was conducted as a cross-sectional study, set in urban metropolis of Karachi (24°53 N and 67°00 E) - the largest city of Pakistan during the period of January 2011. The survey was performed by establishing medical camps, in a time span of three weeks, at two city shopping centers-Defence Sunday Market and Gulshan-e-Iqbal Friday market. These centers were identified due to their central location and variability of attendees from diverse socioeconomic background. A high population density and ethnic diversity within the corresponding areas make both of these sites good representatives of the general adult population of the city. All participants were recruited after taking formal informed consent. A pamphlet briefly describing the function of vitamin D along with its sources and study objectives was distributed in the area to inform people about the research objective of the survey.

### Sample Size

Keeping in view the primary aim of our study, to find the prevalence of vitamin D deficiency and insufficiency in asymptomatic population of an urban centre, the sample size was calculated using the Sample Size Determination in Health Studies software provided by the World Health Organization. The reported collective prevalence of vitamin D insufficiency and deficiency in our region is between 85–98%. . Using an anticipated population proportion of 85%, a relative precision of 0.05 with 95% confidence–the sample size calculated was 272, which allowed us to estimate the prevalence of low levels of vitamin D to within 5% of the true value with 95% confidence. Participation of both literate and illiterate asymptomatic adults was ensured by sending medical students and assistants into the market to inform and explain the purpose of the camp and also through posters and pamphlets printed in local language. Asymptomatic healthy respondents between 30–80 years of age were recruited. Individuals with signs and symptoms of hypo-calcemia, pregnant and nursing mothers and those with diagnosed metabolic bone diseases were not included. By conducting this study in a market setting the house bound and less mobile members of the community were not included since housebound adults cannot come to the market locations because of their co-morbidity status or physical disability. Therefore our methodology enabled us to exclude the less mobile, house bound, ill patients and the results reflect vitamin D levels in apparently healthy asymptomatic adults, as per objective of the study.

### Data Collection

The initial stage of assessment involved administration of an interview based questionnaire, at the site of camp. The interviewers, comprising of two medical students and a single research assistant at home university, were trained and briefed prior to taking the interviews, for the purpose of consistency in data collection. The questionnaire recorded information on age, level of education(degree obtained at last year of school or college), monthly gross income, co-morbidity status(such as diabetes mellitus, hypertension and Ischemic heart disease), diet pattern(including daily milk intake per 250 mL servings), sun exposure (approximate number of minutes per day spent in sunlight) and medication use including oral vitamin D supplementation. The physical assessment performed by the same interviewer included a blood pressure check and anthropometric measurements with a balance scale and stadiometre. Hypertensive respondents in the study are defined as those with a prior diagnosis of hypertension and/or those taking pharmacotherapy to control blood pressure. An elevated spot blood pressure is defined as a one time systolic reading ≥140 mm of Hg and diastolic ≥90 mm of Hg, the cut offs were adjusted in diabetics and CKD patients in accordance with the JNC-7 guidelines. Since the study was conducted in a winter month most of the participants, both males and females, were present in the traditional winter dress covering all the body except the face and hands, therefore no objective assessment of covering was made.

### Blood samples

Venous blood samples of 10 cm^3^ were collected by two expert phlebotomists in three plastic serum tubes for each respondent. The samples were placed in ice boxes at the site of the camp and were sent to the lab in batches. There was a time lag of approximately 60 minutes between venous puncture and serum separation after centrifugation at 3000 bpm. After centrifugation the serum was stored in the laboratory freezer at −20°C, until further analysis. Serum markers measured included 25(OH) vitamin D, intact parathyroid hormone (PTH), calcium (Ca), albumin, creatinine (Cr), phosphorus, and alkaline phosphatase (AP). Measurement of serum levels of 25(OH) vitamin D was done by radioimmunoassay technique with Diasorin SR® kits being used. The venous blood samples for PTH analysis were collected in EDTA plasma collection tubes. We were mindful of the implications of time delay and confident that the time lag of approximately one hour between sample collection and completion of centrifugation, allowed for accurate PTH measurements using the DPC(Diagnostic Product Corporation) Immulite® assay.

### Calculations

Renal eGFR was calculated for 288 subjects, by the four variable MDRD formula using the investigated level of serum creatinine and patient anthropometric measurements as taken during the physical assessment. CKD stages were defined, following the National Kidney Foundation clinical practice guidelines.

### Data Entry and Analysis

The data was entered into a pre-designed data base by two individuals separately using Epi Data Version 3.6.1. Both data sets where then checked to detect any errors in data entry and all discrepancies during data entry, were rectified using the hard copy of the filled questionnaires and laboratory data. The data was analyzed using Statistical Package for Social Sciences version 17 (SPSS v.17.0®).

In uni-variate analysis, descriptive statistics were used to look at the spread of data with respect to the age, education and economic status. Proportion & percentages were computed for categorical variables. Tests for normality, including the Shapiro Wilk test, were used to check for normality assumption for continuous variables. Due to the asymmetric distributions of the outcome variable of vitamin D levels along with other biochemical markers and presence of atypical values (outliers), parametric tests including linear analysis of variance (ANOVA) and linear regression tests were not used. The median and inter-quartile range was used to describe the sample.

The variable of serum vitamin D level was divided into three categories of deficiency, insufficiency and sufficiency, for the purpose of bi-variate analysis. Chi square test was used to find any associations between groups of vitamin D levels and other categorical variables. Non parametric tests including the Kruskal-Wallis test and Mann Whitney U test were conducted to find any significant difference in continuous variables between groups of the vitamin D levels. Continuous relationships of 25(OH) vitamin D levels with various patient characteristics including gender, hypertension status, CKD stage, milk intake and sunlight exposure were investigated using the Mann Whitney U and Kruskal Wallis test. Spearman's rank correlation was applied to determine associations of vitamin D levels with other serum biochemical markers and eGFR. A p-value of <0.05 was taken to be statistically significant.

## Results

Our sample of 300 comprised of 194(65%) males respondents. The median age of the sample was 48 (interquartile range 38–55) years. The minimum age in the sample was 30 years, with a maximum age of 80 years being reported. The respondents came from diverse educational backgrounds with 23.4% holding intermediate (2 years of college) degree and 14% having no formal education at all. The median gross monthly income was USD $172(interquartile range $ 115–344). Table 1 summarizes the sample characteristics as seen in each of the groups of vitamin D deficiency, insufficiency and sufficiency.

**Table 1:** Sample characteristics with respect to Vitamin D groups.

	Deficiency (n = 173)	Insufficiency (n = 80)	Sufficiency (n = 47)	p-value
**Age(years)**	47.0 (35.0–54.0)	48.0 (39.5–56.5)	52.0 (40.8–64.2)	0.021
**Gender**				0.134
**Male(n = 194)**	107(55.2%)	59(30.4%)	28(14.4%)	
**Female(n = 106)**	66(62.3%)	21(19.8%)	19(17.9%)	
**Median Vitamin D levels (ng/dL)**	13.4 (9.9–17.1)	23.5 (21.6–25.6)	34.9 (32.9–41.8)	0.001
**BMI (kg/m^2^)**	27.4 (24.8–30.8)	26.7 (23.9–30.1)	26.5 (23.9–29.9)	0.326
**PTH (pg/ml)**	44.4 (34.3–56.8)	35.6 (26.9–43.9)	38.4 (28.0–48.8)	0.001
**Calcium(mg/dl)**	9.46 (9.24–9.67)	9.44 (8.92–9.68)	9.49 (9.23–9.71)	0.189
**Phosphorus(mg/dl)**	3.14 (2.71–3.43)	3.08 (2.88–3.45)	3.18 (2.78–3.61)	0.846
**Albumin (g/dl)**	4.92 (4.73–5.06)	4.89 (4.57–5.05)	4.80 (4.56–4.97)	0.100
**Alkaline Phosphate (U/L)**	91 (79–106)	95 (74–108)	97 (72–116)	0.819
**Creatinine (mg/dl)**	0.77 (0.67–0.86)	0.78 (0.67–0.93)	0.77 (0.66–0.97)	0.370
**eGFR (ml/min per 1.73 m^2^)**	105.8 (90–119)	101.7 (90–121)	103.0 (82–116)	0.234
**Diagnosed CKD(n = 7)**	4 (57.1%)	1 (14.3%)	2 (28.6%)	0.556
**Hypertension(n = 78)**	45 (57.5%)	19 (24.4%)	14 (17.9%)	0.755
**High spot Bp(n = 97)**	62 (63.9%)	27 (27.8%)	8 (8.2%)	0.078
**Diabetes Mellitus(n = 89)**	54 (60.7%)	19 (21.3%)	16 (18.0%)	0.373
**Vitamin D Supplement use(n = 74)**	36 (48.6%)	19 (25.7%)	19 (25.7%)	0.021
**Daily sunlight >30 mins(n = 239)**	143 (59.8%)	57 (23.8%)	39 (16.3%)	0.092

Median values and inter quartile ranges are reported.

**Figure 1:**
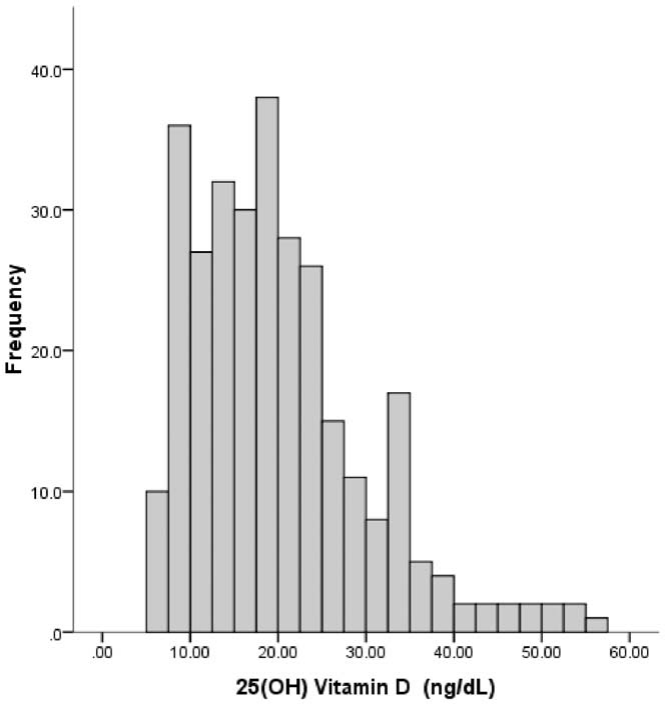
Distribution of 25(OH) vitamin D.

The median level of serum 25(OH) vitamin D was 18.8(interquartile range 12.65–24.62) ng/dL, with sample vitamin D levels ranging from 5.23 to 56.32 ng/dL. A total of 253 (84.3%) respondents had low (<30 ng/dL) levels of vitamin D, comprising of 173(57.7%) respondents in the vitamin D deficiency group. In terms of gender, the median serum vitamin D levels in male respondents came out to be 18.99(interquartile range 12.28–24.41)ng/dL compared to 18.18(interquartile range 13.36–25.00)ng/dL in females. Eighty six percent of the men and 82% of the females were found to have serum 25(OH) vitamin D levels less than the 30 ng/dL cut off for vitamin D sufficiency. Looking at continuous relationships, vitamin D levels in the sample were not significantly different between males and females (p = 0.994). Vitamin D deficiency was more frequently observed in females (62.3%) than in men(55.2%), however this difference was not statistically significant(p = 0.134).The distribution of vitamin D levels in the sample have been shown in Figure 1.

**Figure 2:**
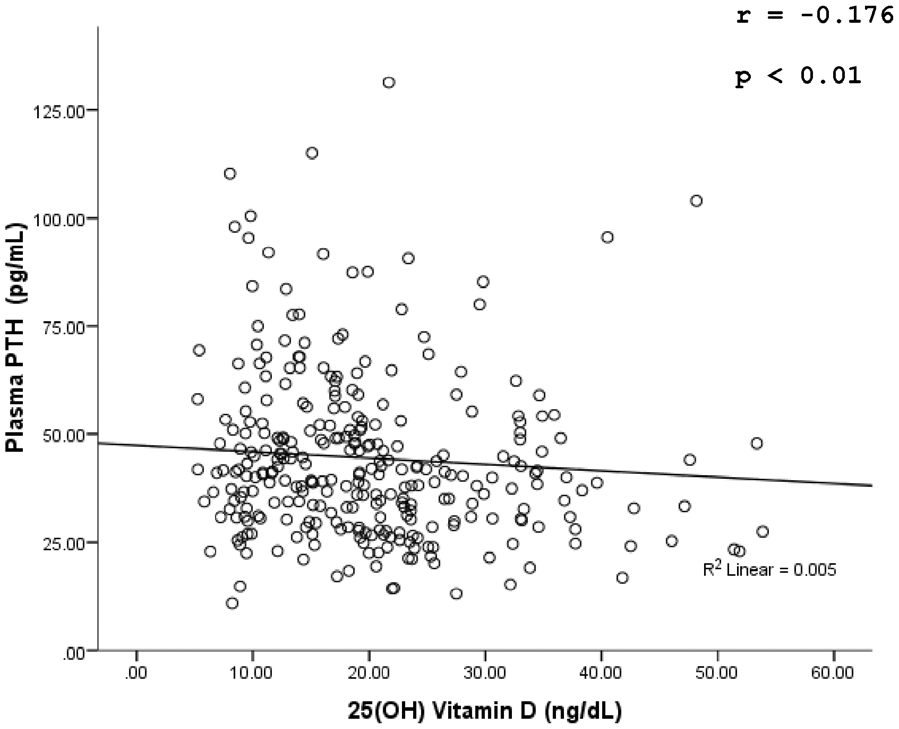
Scatter plot showing negative correlation between vitamin D and PTH.

The PTH levels ranged from 11 pg/mL to 146 pg/mL. A significant negative correlation was observed between vitamin D levels and PTH levels (r = −0.176, p = 0.001), as shown in figure 2. A statistically significant difference was seen in the median PTH levels, when the group of vitamin D sufficiency was compared with the deficiency group (p = 0.011).However there was no significant difference between the sufficiency and insufficiency groups (p = 0.600). Hyper-parathyroidisim (PTH>87 pg/mL) was seen in 14 individuals (4.7%) having a median 25OH vitamin D level of 17.3 (interquartile range 9.78–27.67) ng/dL. Out of the 14 individuals with hyperparathyroidism, nine respondents were found to be having deficient levels of vitamin D with a median serum vitamin D level of 11.35 (interquartile range 9.05–17.30) ng/dL.

The median serum calcium level observed was 9.46 (interquartile range 9.18–9.68) ng/dL. There was no significant difference in serum calcium levels when the vitamin D sufficiency group was compared with deficiency and insufficiency groups (p = 0.636, 0.125 respectively). Serum phosphate levels of lower than 2.5 mg/dL were seen in 41(13.6%) respondents. An elevated phosphate level (>4.5 mg/dL) was seen in four (1.3%) respondents, with all four having vitamin D levels of less than 30 ng/mL.

**Figure 3:**
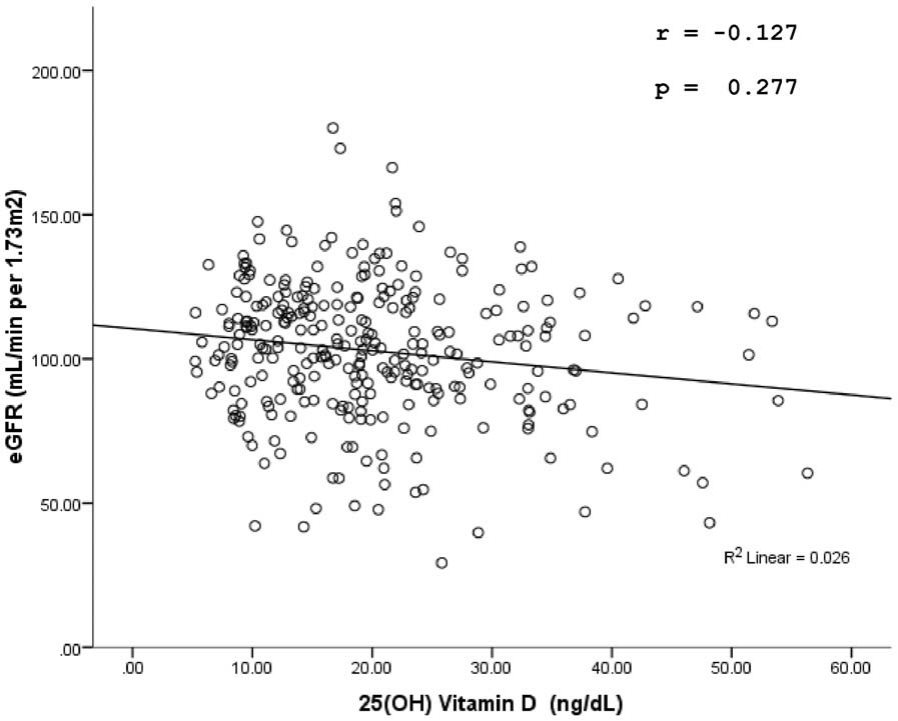
Scatter plot between vitamin D and Egfr.

In terms of calculated GFR we observed no significant correlation with vitamin D levels (r = −0.127, p = 0.277) or difference when the group of vitamin D sufficiency was compared with the insufficiency and deficiency groups (p = 0.316, 0.096 respectively).The continuous relationship between vitamin D and eGFR is shown in figure 3. On the basis of eGFR, no CKD was seen in 213 respondents (75%), 61(21%) respondents had an eGFR corresponding to stage 2 CKD and 14 respondents (5%) had eGFR corresponding to stage 3 CKD. There was no statistically significant difference in the median 25(OH) vitamin D levels between respondents in the three CKD stage groups (p = 0.127).In the vitamin D deficiency group 41 (24%) respondents with vitamin D deficiency had an eGFR corresponding to stage 2 and stage 3 CKD, compared to 16(20%) and 18(38%) respondents from the insufficiency and sufficiency group respectively. As observed 75(25%) respondents had a GFR<90 mL/min per 1.73 m^2^, corresponding to CKD stages 2 and 3, this is in stark contrast with the 7(2.3%) respondents reporting co-morbidity with CKD at the time of questionnaire administration.

Hypertension status had no significant association with vitamin D levels since there was no statistically significant difference in vitamin D levels when respondents with and without hypertension were compared(p = 0.771). Similarly we saw no association between an elevated spot blood pressure and vitamin D levels (p = 0.164).At the time of assessment 78(26%) respondents reported a co-morbidity with hypertension out of which 53(18%) were taking antihypertensive medication. An elevated spot blood pressure was observed in 97 (32%) respondents, out of which 22(23%) respondents were taking antihypertensive medication while 61(63%) respondents with an elevated spot blood pressure had no prior diagnosis of hypertension. At least one daily milk serving (250 ml) and daily sunlight exposure greater than 30 minutes were not found to have an significant association with vitamin D levels, as depicted by p-values of 0.618 and 0.279, respectively.

The regular use of oral vitamin D containing supplements on a daily basis was significantly associated with vitamin D levels (p = 0.021).In the vitamin D deficiency group, it was observed that only 36(21%) respondents used vitamin D containing supplements compared to 19(40%) in the sufficiency group. Overall in the sample of 300, only 74(24.7%) respondents reported use of oral vitamin D containing supplements with 59(19.6%) respondents taking one dose on a daily basis.

## Discussion

As seen in our study low levels of vitamin D (<30 ng/dL) were present in 84.3% of the general population with both genders affected equally. This finding, although expected, in itself is very significant and considering the fact that our study sample comprised of asymptomatic adults in the community, it does raise a health care concern that a sizeable majority of the healthy population is deprived of the proven benefits of vitamin D. The overall figure of 84.3% respondents having low vitamin D levels is comparable to findings reported by other studies done in Karachi on vitamin D levels, however we observed that 15.7% of respondents had sufficient levels(>30 ng/dL) of vitamin D compared to 8.9% and 8.0% reported previously by Mansoor et al. and Zuberi et al. respectively [17], [22]. The larger size of the sufficiency group as seen in our results may be explained by the difference in study setting, with both the above mentioned studies being conducted in a hospital setting in contrast to the community setting employed in our study. In our study, 57.7% of the respondents had vitamin D deficiency(<20 ng/dL), this is lower than 79.6% and 69.0% prevalence of vitamin D deficiency reported in studies conducted on general asymptomatic population in Tehran [25] and Tripurti, South India [26] respectively. Again these differences in results from regional studies prove even though vitamin D deficiency is prevalent region wide, the proportion of population affected varies.

Despite the observational nature of our research and inability to identify predictors of vitamin D status, we feel it is important to discuss the possible causes of low vitamin D levels seen in the majority of our sample. Amongst dietary factors contributing to low vitamin D levels is the lack of a food fortification policy in Pakistan and a traditional diet lacking in vitamin D. More over the beneficial role of vitamin D supplementation is underutilized in the community as only 74(24.7%) respondents reported use in our results. Darker skin pigmentation has also been associated with decreased skin synthesis of vitamin D [4], [27] as have been habits of betel and areca nut chewing [28] which is a common practice in Karachi specifically, though these factors have not been accounted for by our study.

The blood samples were taken in the month of January, which corresponds to winter season in Karachi. The average day length in January has been estimated to be 10.6 hours compared to 13.5 hours in May. Also the average total monthly sunshine hours in Karachi, during January (271 hours), is less than the summer months of April (282 hours) and May (304 hours). Keeping in view the shorter day length and decreased number of total sunshine hours, it can be logically concluded that respondents in Karachi have lower sun exposure in January as compared to the summer months. The seasonal variation in levels of serum 25OH vitamin D due to the differences in sunlight exposure was studied by Goswami et. al in a prospective study done in Delhi, which showed that the study group had significantly higher 25 OH vitamin D levels in summer as compared to the winter assessment [29]. Similarly Azizi et. al. found out that vitamin D sufficiency was a more frequent finding in the summer months than in winter, amongst the working male population of Tel Aviv, Israel [30]. Therefore low sun exposure in January and preceding winter months, may explain the high proportion of respondents having vitamin D deficiency (57.7%) and insufficiency (26.6%) we see in our study.

In our results, vitamin D was negatively correlated to PTH levels (r = −0.176) and the PTH levels were significantly higher in the vitamin D deficiency group when compared with the sufficiency group. Despite a high number of individuals being deficient in vitamin D(57.7%), the number of respondents having hyperparathyroidism(4.7%) was less than expected-this observation is in contrast to the results seen by various authors [17], [22], [25], [31]. Although, secondary hyperparathyroidism has often been used a marker for vitamin D deficiency [32] in our study this was not observed. This may be explained by the fact that 13.6% of the sample had low phosphate levels. Serum phosphate has been found to have a direct effect on PTH secretion, independent of ionized calcium and 1,25(OH)vitamin D, via post-transcriptional mechanisms [33]. Therefore low serum phosphate levels may have been a cause for decreased stimulus for PTH secretion and hence the lesser number of respondents with secondary hyperparathyroidism.

The favorable effects of vitamin D supplementation have been documented by Pelicane et. al. [34] in a retrospective study and were also seen in our study with 40% of the respondents in the sufficiency group reporting use of vitamin supplements containing vitamin D compared with only 21% in the deficiency group. In contrast to our findings in the general population Thomas et. al. found multivitamin therapy was not protective against hypo-vitaminosis D in medical inpatients [35], however the study being mentioned was done in a setting of acutely ill patients. According to the 2011 Institute of Medicine (IOM) report, the recommended daily intake for vitamin D, for adults between 19–70 years of age is 600 IU/day for achieving adequate bone health. There is still debate on whether the daily intake should be increased to 1500–2000 IU/day to maintain serum 25(OH) vitamin D levels above the desired 30 ng/dL mark [18]. Similarly with increasing age and in countries where sun exposure is inadequate the recommended intake should be a minimum of 800–1000 IU/day [36]. Considering the fact that the daily requirement for vitamin D is barely met by dietary intake and limited sun exposure, the need and benefit of supplementation is well established in international literature, but no local or regional guidelines are available on the subject.

Chronic kidney disease patients have a higher propensity for developing 1,25(OH)vitamin D deficiency and it is almost a universal finding in patients with end stage renal disease [8], [37]. There have also been findings of an increased mortality risk in advanced CKD patients having deficient serum levels of 25(OH) vitamin D [38]. In our study no correlation was seen between estimated GFR and serum vitamin D levels, this could be due to the reason that we did not measure serum 1,25(OH) vitamin D levels. The more surprising finding was that 25% of the study population had an estimated GFR corresponding with CKD stage 2 and stage 3.Viewing this with the fact that only 2.3% of the sample reported of a co-morbidity with CKD, we feel that future studies that assess sub-clinical deterioration of renal function in natives of this region are warranted.

### Conclusion

We observed a high proportion of the asymptomatic general population having low levels of vitamin D and subclinical deterioration of eGFR. The possible causes of low levels of serum 25OH vitamin D seem to be multiple and need to be investigated in further detail in order to address this public health concern.

### Limitations of study

Our inability to draw a random sample may reduce the generalize-ability of results. Since the difference between demographics and co-morbidity status of asymptomatic responders and non responders is not known, there is a potential for a selection bias. The cross sectional design of our study with a single blood sample collection in a winter month, having low sun exposure, and application of non parametric analysis proves to be a limitation in drawing concrete conclusions about a population therefore a longitudinally designed research proposal with serial blood sampling performed in both the winter and summer months is recommended. Increasing sample size with inclusion of more pockets of population including rural population would help in generalize ability.
